# Alveologenesis: What Governs Secondary Septa Formation

**DOI:** 10.3390/ijms222212107

**Published:** 2021-11-09

**Authors:** Alexandra L. Rippa, Elena V. Alpeeva, Andrey V. Vasiliev, Ekaterina A. Vorotelyak

**Affiliations:** Laboratory of Cell Biology, N.K. Koltzov Institute of Developmental Biology, 26 Vavilov Str., 119334 Moscow, Russia; alpeeva_l@mail.ru (E.V.A.); 113162@bk.ru (A.V.V.); vorotelyak@yandex.ru (E.A.V.)

**Keywords:** alveologenesis, secondary septa, alveolar interstitial resident fibroblasts, myofibroblasts, lipofibroblasts, retinoic acid signaling, extracellular matrix, elastin, lung regeneration, pneumonectomy

## Abstract

The simplification of alveoli leads to various lung pathologies such as bronchopulmonary dysplasia and emphysema. Deep insight into the process of emergence of the secondary septa during development and regeneration after pneumonectomy, and into the contribution of the drivers of alveologenesis and neo-alveolarization is required in an efficient search for therapeutic approaches. In this review, we describe the formation of the gas exchange units of the lung as a multifactorial process, which includes changes in the actomyosin cytoskeleton of alveocytes and myofibroblasts, elastogenesis, retinoic acid signaling, and the contribution of alveolar mesenchymal cells in secondary septation. Knowledge of the mechanistic context of alveologenesis remains incomplete. The characterization of the mechanisms that govern the emergence and depletion of αSMA will allow for an understanding of how the niche of fibroblasts is changing. Taking into account the intense studies that have been performed on the pool of lung mesenchymal cells, we present data on the typing of interstitial fibroblasts and their role in the formation and maintenance of alveoli. On the whole, when identifying cell subpopulations in lung mesenchyme, one has to consider the developmental context, the changing cellular functions, and the lability of gene signatures.

## 1. Introduction

The postnatal development of alveoli is the most important and less investigated phase of lung development. The pathology of alveolar formation is often detected during the occurrence of pulmonary diseases in adults and children. However, the mechanisms of alveologenesis remain mostly understudied.

Alveologenesis is the final stage of lung maturation, when an alveolar region is divided into smaller units called alveoli via the process known as secondary septation. Each of the formed septa serves as a new gas exchange surface, and all together, they dramatically increase the respiratory surface area. The disruption of alveologenesis leads to the simplification of the alveoli, as seen in preterm infants diagnosed with bronchopulmonary dysplasia (BPD)—a widespread pulmonary disease that is often connected with lifelong respiratory failure. There are approaches that can be used to study lung development, including alveologenesis. Among them is the cultivation of cells and lung slices in vitro. Pieretti et al. showed that P4 lungs inflated with agarose and grown for four days, embedded in a collagen matrix, appear to septate similarly to lungs that grow in vivo [1]. Live images of alveologenesis in precision-cut lung slices can reveal dynamic cell behavior [2]. Lung organoids and lung-on-a-chip techniques can also serve as models for the investigation of lung development, functions, and respiratory diseases [3,4]. Much information about signaling pathways has been obtained using mouse models with cell-specific gene ablations, some of which will be described here. In vivo models of alveoli regeneration such as lung regeneration after an injury caused by exposure to smoke or chemicals or the realveolarization after partial pneumonectomy (PNX) also play an important role in understanding pulmonary alveologenesis. The development of alveoli is regulated by a complex network of signaling pathways, which involve different pulmonary cell types. Lung mesenchyme cells, such as alveolar myofibroblasts [5], and also endothelial cells [6] are considered to be drivers of alveolar development. Epithelial–mesenchymal interactions are known to regulate lung morphogenesis, while the mesenchyme possesses induction activity [7,8] and consists of multiple cell subpopulations, which differ in spatial arrangement and functions. Among them are airway and vascular smooth muscle cells; pericytes; interstitial myo-, matrix-, and lipofibroblats; the recently revealed myofibrogenic progenitor cells, which preferentially generate abnormal myofibroblasts after injury; alveolar niche cells necessary for the maintenance of epithelial proliferation and differentiation [9]; and mesenchymal stem cells, the rare and poorly described population of progenitors with proliferative potential that are activated after injury and are able to differentiate in several mesenchymal cell types. Identifying origin distinctions of fibroblast-like cells is a very difficult task. The activation of ligands and receptors of the main pathways used for clone tracing in the mesenchyme strongly depends on context. Despite the availability of single-cell RNA sequencing (scRNAseq) data allowing for the identification of the profile of mesenchymal lung cell subpopulations, isolating these cells by their characteristic markers remains difficult, given that their functionality and heterogeneity changes during development. One single signaling pathway can be activated in multiple fibroblast subpopulations. Cells repeatedly activate the same signaling pathways during lung development and regeneration after injury; however, at the same time, they can function as rather distinct clones. Thus, the moment of activation of the lineage marker (signaling pathway molecules) plays an important role in progenitor identification and the determination of its lineage trajectory [10]. Cell subpopulations are rather thought to be transient states, common for this or that subtype of interstitial fibroblasts than definite cell types. In adults, pulmonary diseases such as chronic obstructive pulmonary disease (COPD) and lung fibrosis are connected with the impairment of alveolar regeneration. Despite the apparent variety of studies and literature on lung development, specifically postnatal development, and the use of modern techniques such as scRNAseq, the data on alveologenesis are rather scattered and do not provide a holistic idea of the complex of cellular, mechanical, and molecular interactions that ensure the formation and regeneration of alveoli. In this review, we tried to elucidate the results of the latest research on alveologenesis, including septal formation and the role of mesenchymal cells supporting it, and to arrange information and identify gaps in this area in order to ensure the effective elaboration of methods for eliminating developmental disorders and ensuring the normal functioning of the lungs. In the first instance, we were interested in the analysis of scattered data on alveologenesis and realveolarization: the variety of lung mesenchymal cells, elastogenesis, mechanics, alveolar shaping, and the contribution of septal elements (alveolocytes, extracellular matrix (ECM), blood vessels) to these processes. We also intended to pinpoint the importance of comparing the functions of mesenchymal cells with their morphotypes and molecular signatures in alveologenesis. Nevertheless, these identities have not been matched, and it makes the search for the ways and targets that influence postnatal alveologenesis, and thus the regeneration processes, more complicated. The discussed model of PNX is widely used for the investigation of the processes of reparation and regeneration because regeneration, in this case, is enabled via the compensatory lung growth, which provides the possibility of studying the mechanisms of the reinitiation of alveolar septum formation (septation) more profoundly.

The data discussed in this review were obtained mostly in mice experiments; however, in some cases, especially regarding pathology, we refer to human studies and specify this.

## 2. Briefly about Embryonic Lung Development

**Figure 1:**
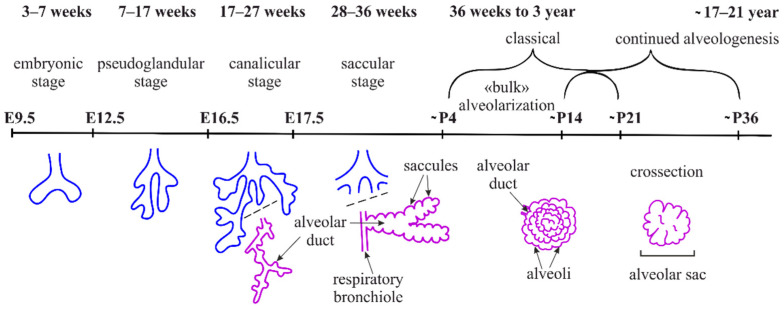
Lung development. Development of the lung as a whole and its proximal parts is shown in blue. Development of the distal lung and alveologenesis is shown in purple. E, embryonic day; P, postnatal day. The timescale, shown in weeks, refers to the stages of human lung development.

Lung development in mice begins at around E9.5. It can be divided into the pseudoglandular (E10.5–E16.5), canalicular (E16.5–E17.5), saccular (E17.5–P5), and alveolar (P3–P30 (P39)) phases according to morphological features (Figure 1).

In the developing fetal lung, the interplay between the epithelium and the mesenchyme surrounding it is a driving force that guides the formation of the proximal–distal pattern. Respiratory mesoderm originates from the cardio-pulmonary precursors expressing Wnt2, Gli1, and Isl1 and generates vascular and airway smooth muscle cells, proximal vascular endothelium, and pericyte-like cells [11]. Tbx4 is expressed in the population of the mesenchymal cells that give rise to the entire stroma of the postnatal lung. TBX4-positive mesenchymal precursor cells proliferate and differentiate into different cell types, simultaneously cooperating with the developing lung epithelium [12,13]. In the regions adjacent to the endoderm of the distal tip, mesodermal cells express FGF10, which stimulates branching morphogenesis [14,15,16,17]. In the fetal and postnatal lung, FGF10^+^ cells represent the pool of mesenchymal progenitor cells [18]. The endoderm of the distal tip expresses SOX9 and ID2. Endodermal cells respond to WNT and the chemoattracting and morphogenic action of mesodermal FGF10 (for the review, see [19]). The Shh and Ptch1 expression patterns are the reverse of the expression patterns of Fgf10 and Fgfr2b in that the Shh ligand is expressed in the epithelium, which is of endodermal origin, while the Ptch1 receptor is expressed in the mesenchyme [20]. The formation of a vast branching network of airways is partly explained by a signaling mechanism based on a ligand–receptor interplay between Fgf10 and Shh, which directs the outgrowth of the lung bud via a ligand–receptor-based Turing mechanism, and is additionally determined by a geometry effect of the lung [21]. The lung mesenchyme possesses positional information and anatomical specificity: proximal and distal mesenchyme can induce the corresponding proximal and distal differentiation of the lung epithelium [7,8]. In 1994, Shannon was already able to show the induction of alveolar type II cell differentiation in fetal tracheal epithelium by the transplanted distal lung mesenchyme [7].

During the pseudoglandular stage, the recurrent processes of outgrowth and branching of the lung endoderm (branching morphogenesis) are initialized, by which all the future respiratory airways (bronchi and bronchioli) are formed (Figure 1). It is interesting to note that the specification of alveolocyte clones occurs earlier than it was supposed before; more specifically, it runs in parallel to the processes of tissue patterning, including the formation of the axial endoderm pattern and branching morphogenesis [22]. At the end of branching morphogenesis on E15.5, precursors of the alveolar type 1 and 2 cells (AT1 and AT2) are detected in the bud tips [5]. During the canalicular phase, the differentiation of the epithelium can be detected (via alveolocyte marker staining), and the junctions of bronchiolar ducts, which are the extensions of the conducting airways, are formed. Further in development, the enlargements (saccules) are formed along the branching tree, thus marking the saccular phase (Figure 1). Separations between initial alveolar sacs are known as primary septa. Several authors also designate this phase as initial alveologenesis. Primary septa are not effective in the formation of the air–blood barrier and full breathing. Thus, the survival of an individual depends on the timely and accurate formation of the secondary septa. Mice are born in the saccular phase of lung development. The formation of their secondary septa and alveoli occurs after birth. In humans, alveologenesis begins in utero on the 36th week of gestation (Figure 1), and classical alveologenesis continues for 2–3 years after birth, when the number of alveoli increases. Afterwards, from 3 until 21 years, the size of alveoli increases [23].

## 3. Postnatal Alveologenesis

**Figure 2:**
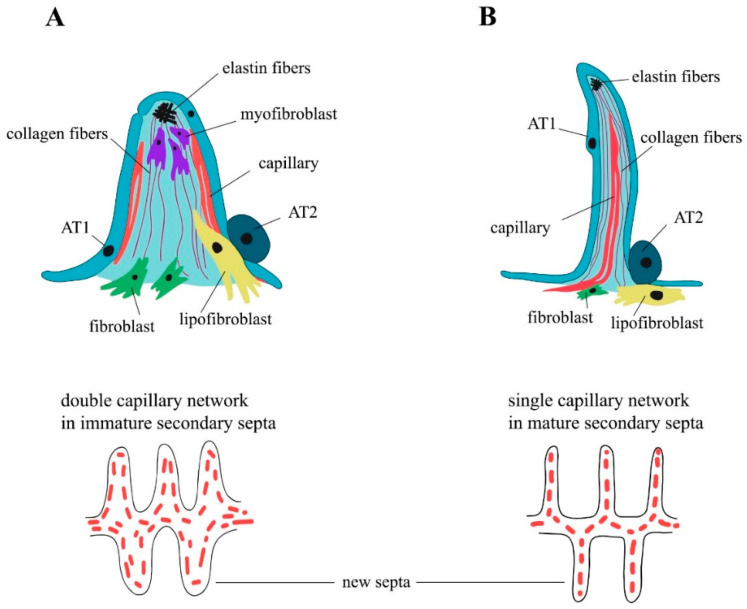
Alveolar secondary septa. Classical alveologenesis. Immature, thick secondary septa. Double capillary network (**A**). Continued alveologenesis. Mature, thin secondary septa. Single capillary network (**B**). Redrawn from [23,26].

The formation of the alveoli (alveologenesis) is the final stage of lung development and is subdivided into two phases (Figure 1). The first phase begins on the 3rd–4th day of postnatal development (P3–P4), and is characterized by the formation of the secondary septa, which represent the invaginations of the saccular walls rich in ECM and separate and expand the alveolar surface area [24]. Around P3, as the breathing mode is established, a subpopulation of the secondary crest myofibroblasts (SCMFs, also known as alveolar myofibroblasts) with increased traction force appears in the lung mesenchyme [5]. SCMFs participate in secondary septa formation and actively accept different signals from AT1 cells. Contractile activity of myofibroblasts is supposed to physically form alveoli [25]. SCMF precursors are the subpopulation of platelet-derived growth factor receptor α (PDGFRα) positive cells that later in embryonic development become α-smooth muscle actin (αSMA) positive and are found on the tips of the alveolar septa in close proximity to deposits of elastin (Figure 2A).

PDGF-A and its sole receptor PDGFRα constitute an axis of cross-communication between pulmonary endodermal and mesodermal cells [27]. Endodermal cells express the ligand [27], and PDGFRα is ubiquitously expressed throughout the lung mesoderm [28,29]. Earlier, PDGF-A signaling over PDGFRα was shown to be necessary for lung growth and alveoli formation, but not for branching morphogenesis [30]. According to a widespread model of septa formation, PDGF-A secreted by the epithelium is extruded into the lumen of alveoli by the exerting force of myofibroblasts and developing capillary. Then, the septa are thinned through the processes of apoptosis and capillary maturation [31,32]. Impaired Notch signaling in mice, especially Notch2, results in the abnormal enlargement of the alveoli. During neonatal life, Notch2 is activated in AT2 cells to induce PDGF-A expression, triggering the paracrine activation of PDGFRα signaling in the SCMF precursors required for alveologenesis [33]. The first phase of alveologenesis, from around P5 to P14, when SCMFs are present, is called classical alveologenesis (Figure 2A). During the second phase of alveologenesis (continued alveologenesis), from around P14 to P36, formed immature septa start thinning, the double capillary septal network is modified to a single one, thus forming mature, thin septa in an adult lung (Figure 2B) [32]. After the active phase of alveologenesis is completed, more than 20% of interstitial fibroblasts are subjected to apoptosis, a process that is crucial for the normal development of the lung [34,35]. Nevertheless, taking into account the cell heterogeneity of the mesenchymal compartment, the question of which subpopulation of fibroblasts is eliminated thus becomes controversial [34,35,36]. There is an opinion that the thinning of the alveolar walls during the second phase of alveologenesis is caused by the depletion of lung myofibroblasts after around P15 through apoptosis and phagocytosis [37]. The flow cytometry evaluation of lipid-filled interstitial fibroblasts stained with a lipophilic marker showed that this exact subpopulation is eliminated via apoptosis after alveolarization [38]. Results of several studies have suggested the temporal expression of αSMA by some subpopulations of alveolar mesenchymal cells that play the role of SCMFs and are not eliminated afterwards [39,40]. Multiple data have demonstrated that myofibroblasts are crucial for secondary septa formation, but the mechanisms of αSMA^+^ SCMFs depletion after the first phase, and the question of which cells and mechanisms participate in the second phase of alveologenesis after the elimination of αSMA^+^ SCMFs by P15, are the subject of future investigations.

Alveolar density reaches its peak on P39 and stays unchanged after 9 months. The generation of alveoli is fast between P5 and P14, but slows down afterwards [41]. As mentioned before, immature secondary septa have a double capillary network and are quite ineffective in the required gas exchange (Figure 2A). During the maturation of the blood microvessels, the septa start thinning and two capillary layers merge and thus form a more effective single capillary network (Figure 2B) (for the review, see [23]). The single capillary network exchanges oxygen and carbon dioxide with two adjacent alveoli, which adds to the efficacy of the gas exchange. On P4–P21, new secondary septa are formed from immature secondary septa emerging at first from the walls of saccules, and further in development from the walls of immature secondary septa with the double capillary network. On days P14–P36, new septa are lifted off mature septa containing single capillary networks (Figure 2). At the same time, the local duplication of the capillary network due to angiogenesis is found at the base of the newly formed septa in the second phase of alveologenesis. The alveolarization and maturation of microvessels are the processes that run in parallel [42]. Lung development continues into young adulthood: for mice, data are known up to P39 [41], for rats, up to P60 [43]. Alveoli are, in fact, able to form at any time and anywhere inside the lung parenchyma [23]. Thus, in the alveolar septum, the air–blood barrier has thick parts that contain the nuclei of AT1 cells and fibroblasts and the fiber network of ECM, which provides the regenerative capacity and mechanical stability of the septum; it also has thin parts where the alveolar epithelium and the capillary endothelium share one common basal lamina that minimize the thickness of the diffusion barrier. In the human lung, about half of the entire air–blood barrier is thin [44,45].

## 4. Diversity and Origin of Alveolar Interstitial Resident Fibroblasts

During development, alveolar fibroblasts are usually divided into four functional subpopulations: myofibroblasts (SCMFs), matrix fibroblasts, lipofibroblasts, and mesenchymal alveolar niche cells (also known as MANCs), which partially overlap in expressed markers, origin, and activity (Table 1). Based on localization and PDGFRα expression, they are grouped into a pool of interstitial resident fibroblasts (iReFs) [10,46]. It is important to note that this division is not always specific since the expression of certain markers by certain fibroblasts directly depends on the context and the moment of development. Many researchers do not specify which fibroblasts they studied, calling them either “fibroblasts” or “interstitial fibroblasts” or “myofibroblasts”, although it is obvious that this group was heterogeneous in their studies. Moreover, scRNAseq methods have found widespread use only recently, and it has become possible to identify even more subpopulations in each of these groups and trace their origin; however, this sometimes complicates the identification of fibroblasts according to their activity and function. Therefore, at the moment, there is a gap between the molecular classification of iReF subpopulations, morphology, and the precise definition of their functions.

In simple terms, one can say that as septa mature, SCMFs produce elastin, contract and ensure the elongation of the septal tips, SCMFs and matrix fibroblasts secrete metalloproteinases and ECM-forming and remodeling proteins to thin the septal tips and ensure structural support, while lipofibroblasts support AT2 cell surfactant production. At the end of alveolarization, adult alveolar niche stem cells become defined, consisting of the lipofibroblast-like MANCs, which support alveolar growth and regeneration [9,19,26,38,59,63,64,65]. Recent lineage tracing and RNA sequencing (RNAseq) data identified Lgr5^+^ and Wnt-responsive/PDGFRα^+^ mesenchymal cell subsets within alveolar niches that influence the differentiation of alveolar epithelial cells during repair of the mature lung [59,65]. It is now obvious that information on the isolation of subpopulations within the fibroblast population according to the network of activated signaling pathways has to be placed in correspondence with fibroblast classification using markers.

SCMFs and alveolar lipofibroblasts are the best described of these four subpopulations in alveologenesis, as they play a primary role in secondary septation. Alveolar SCMFs are interstitial contractile cells that generate mechanical force to lengthen the tip of the septum, synthesize elastin-rich ECM, express PDGFRα and the smooth muscle marker αSMA encoded by Acta2; they are located on the tips of the developing septa and are embedded in elastin fibers (Figure 2A) [27,39]. In contrast to myofibroblasts, alveolar lipofibroblasts are located at the base of developing septa, are less associated with elastin fibers, and differ markedly in morphology, having fewer secretory organelles and extensive accumulations of intracellular lipids [66]; nevertheless, they also express PDGFRα (Figure 2A,B) [47,63]. The expression of the adipose differentiation-related protein (ADRP encoded by Plin2) enables lipofibroblasts to take up, store, and subsequently transfer triglycerides to neighboring AT2 cells that are to be incorporated into surfactant phospholipids and protect alveoli against oxidative injury [67,68]. ADRP is used as a marker for lipofibroblasts; however, the identity of lipofibroblasts, as determined by the ADRP marker, is still debated since, based on transcriptomic and genetic analyses, this marker has been shown to be widely and nonspecifically expressed [69]. For example, it was detected in macrophages and non-mesenchymal cell populations, presumably hematopoietic-derived cells [70]. Lipofibroblasts are the major storage of vitamin A in the lungs, mainly in the form of retinyl esters. In addition to the synthesis of triglycerides and retinoids, lipofibroblasts are a component of the niche of progenitor AT2 cells and support their proliferation and differentiation into AT1 cells [57,68,71,72,73].

As noted above, all iReFs express PDGFRα. PDGFRα-PDGF-A signaling controls cell proliferation, survival, and cell migration during lung development and regeneration. During the development of the lung alveoli, cells expressing PDGFRα are required for secondary septation and exhibit different phenotypes: either lipid-accumulating or expressing high levels of αSMA. Using the constitutive and a conditional PDGFRαCre line, it was observed that the committing of PDGFRα^+^ cells that generate alveolar myofibroblasts (including tip cells of the secondary crests) and lipofibroblasts occurred before secondary septation [58]. At the beginning of alveologenesis, there were two subpopulations of “dim” and “bright” PDGFRα^+^ fibroblasts in the lungs. PDGFRα^+^ GFP bright CD34-expressing cells with neutral lipids were identified as lipo/matrix-associated fibroblasts, whereas PDGFRα^+^ GFP dim αSMA- and CD29-expressing cells behaved like myofibroblasts. The study showed that PDGFRα kinase activity promoted the formation of an alveolar septum during realveolarization after partial pneumonectomy (PNX) by activating the second of these two groups of fibroblasts [46]. How PDGFRα^+^ alveolar cells choose whether to become myofibroblasts or lipofibroblasts is not fully understood. One theory is based on the difference in the expression level of the Pdgfra gene in iReFs. It was observed that the accumulation of lipid droplets is more specific for the cells with a lower expression of Pdgfra [47]. Thus, on P8, neutral lipid droplets predominated in lung fibroblasts with a lower level of Pdgfra expression, while transgelin (tagln), the gene that encodes an actin-binding protein, an early marker of smooth muscle differentiation that belongs to the calponin family, was predominantly expressed in fibroblasts with a higher expression of Pdgfra. PDGF-A increases Sox9 expression in primary lung fibroblast cultures, hence active PDGFRα signaling is required to maintain Sox9. As alveologenesis and septation progress from P8 to P12, fewer lung fibroblasts express PDGFRα and Sox9, while lung fibroblasts containing myocardin-like transcription factor-A increase in number, indicating that Sox9 decreases as fibroblasts become myofibroblasts. Thus, PDGFRα signaling has divergent effects during the differentiation of pulmonary fibroblasts into lipid storage or myofibroblast-type cells [74]. The permanent activation of PDGFRα in fibroblasts leads to fibroblast hyperplasia and increased septal thickness [75]. This may indicate that there is a relationship between PDGFRα receptor signaling and the number of pulmonary fibroblasts carrying this receptor in the lung.

The origin of the SCMF subpopulation is being actively studied. As we have already mentioned, lung mesenchyme is the recipient of Shh signaling. The glioma-associated oncogene homolog 1 (Gli1) family of zinc-finger transcription factors is the nuclear mediator of Shh. Gli1-expressing cells were shown to be precursors of peribronchial and perivascular smooth muscle cells, and many types of lung fibroblasts, including alveolar myofibroblasts and other fibroblasts derived from mesoderm [76]. The precursor SCMFs are committed early in lung morphogenesis, and their descendants Gli1^+^αSMA^+^ SCMFs are present in the growing tips of the alveolar septa [76]. In their work, Li and colleagues conclude that Shh signaling is activated in early myofibroblast progenitors expressing Gli1-cre**^ERT2^**. Wnt signaling is an important regulator of early lung development (for a review of Wnt signaling in lung development, refer to [77]). During myofibroblast differentiation, Wnt signaling controls the size of the progenitor pool, and it is suggested that during the saccular stage of lung development, PDGF-A signaling is required for the migration and distribution of SCMF progenitors in the lung interstitium in order to enable the formation of secondary septa. As a result, the population of differentiated myofibroblasts is localized in the area of the primary septa and the tips of secondary septa and expresses ACTA2 and PDGFRα [76]. As Wnt signaling is also a critical pathway for self-renewal and the specification of stem cells in multiple organs [78], it is interesting to note that its re-emergence during alveologenesis provides a wave of AT2 cell proliferation in the final stages of lung development. Activating Wnt signaling results in the expansion of AT2 cells, whereas the inhibition of Wnt signaling inhibits AT2 cell proliferation and switches alveolar epithelial development to the AT1 cell lineage [79]. The inhibition of Shh signals at the early stages of alveolarization leads to increased lung elasticity, alveolar defects, and a decrease in the number of secondary septa. Shh signaling is also required for myofibroblast differentiation. The inhibition of Shh during early alveolarization almost completely eliminates Gli1^+^αSMA^+^ cells in the septal tips, and Gli1 line tracing showed that Gli1^+^ cells did not undergo apoptosis after Shh inhibition and remained in the alveolar septa, but could not express αSMA. Shh signaling is vital for mesenchymal proliferation during alveologenesis because the inhibition of Shh reduces the proliferation of Gli1^+^ cells and their progeny [40]. The postnatal inactivation of Pdgfra in Gli1^+^ SCMFs results in the arrest of alveologenesis, which is similar to what is observed in the BPD. Elastin mRNA level was decreased, and its distribution was abnormal [80]. As the scRNAseq data showed a coexpression of Gli1, Pdgfra, and insulin-like growth factor type 1 receptor (Igf1r) in a subset of lung fibroblasts on P1, He et al. investigated the consequences of Igf1r deletion in these cells in the postnatal period. They found that this caused alveolar simplification, elastin network disruption, and ECM deposition without altering myofibroblast differentiation or proliferation. Moreover, loss of Igf1r inhibited the contractile activity of myofibroblasts and myosin light chain phosphorylation. IGF1R signaling was required for the activation of PI3K/AKT and YAP activity [81]. In the case of Gli1, it was shown that Gli1^+^ precursors are committed exclusively for the fate of myofibroblasts on E10.5–E11.5, which can also subsequently give rise to a certain population of lipofibroblasts [76]. Nevertheless, the main population of stromal cells containing lipid droplets appears in the developing lung between E15.5 and E16.5 [18]. This is accompanied by the significant activation of the peroxisome proliferator-activated receptor γ (PPARγ), ADRP, and FGF10 in lung mesenchyme, which mark a subpopulation of lipofibroblast progenitors [18]. Fgf10 signaling plays an essential role in the formation of lipofibroblasts during late lung development. The knockdown of the Fgfr2b ligand activity and the decrease in Fgf10 expression in vivo lead to a global decrease in the expression levels of lipofibroblast markers on E18.5 [82]. Park and colleagues revealed the role of the transcription factor Tcf21 in lipofibroblast specification [54]. They showed that after E15.5, Tcf21-expressing progenitor cells mainly become lipofibroblasts and, to a much lesser extent, other iReFs. Thus, lipofibroblast specification is suggested to occur during the saccular phase in Tcf21-expressing cells, and these cells no longer act as precursors of myofibroblasts or smooth muscle cells. Lipid metabolism genes are highly expressed in perinatal and adult Tcf21-traced lineage cells. The overexpression of Tcf21 in primary neonatal lung fibroblasts leads to an increase in intracellular neutral lipids, thus confirming the regulatory role of Tcf21 in lipofibroblast functioning [54].

Another marker of the lipofibroblasts is CD90 (Thy-1). A distinct population of lipid-saturated iReFs is known to express it. Thy-1, a glycophosphatidylinositol-linked cell-surface glycoprotein, activates lipofibroblast differentiation via PPARγ, which causes an increase in fibroblast triglyceride content via fatty-acid transporter proteins [53]. The loss of pulmonary fibroblast Thy-1 expression causes a lipofibroblast to myofibroblast transfer. Lipofibroblasts converted to myofibroblasts are unable to maintain pulmonary epithelial cell growth and differentiation, resulting in impaired alveolarization, which is specific for BPD, fibrosis, and other chronic lung diseases [68,83]. CD90^−^ fibroblasts show higher levels of PDGFRα expression. They show increased proliferation in response to PDGF-A as compared to CD90^+^ fibroblasts [84], which is consistent with the data on different levels of PDGFRα expression in lipofibroblasts and myofibroblasts. Previously, Thy-1^−/−^ mice demonstrated the attenuation of alveolarization with a concomitant increase in TGF-beta signaling, fibroblast proliferation, and collagen and elastin deposition [85]. In addition, hypoxic conditions reduced the Thy-1 mRNA level, while in Thy-1^−/−^ mice treated with the TGF-beta neutralizing antibody 1D11, the development of alveoli and lung function were improved. Thus, it is likely that hypoxia resulting from the decrease of Thy-1 activates TGF-beta and thereby inhibits the normal development of alveoli [85]. Not only Thy-1 but also leptin acts as a modulator of PPARγ; however, its effect is exerted via the parathyroid hormone-related protein (PTHrP). A high fatty diet increases the plasma levels of leptin, which then acts on AT2 cells and increases the mRNA steady-state level of PTHrP [86]. PTHrP acts in a paracrine way on alveolar lipofibroblasts via the stimulation of the PTH-1 receptor-mediated cAMP pathway, which then induces the expression of ADRP and PPARγ. Both these factors improve lipid uptake, the formation of surfactants, and decrease the transdifferentiation of lipofibroblasts into myofibroblasts [87]. Furthermore, lipofibroblasts themselves produce leptin that acts as a positive feedback modulator on the expression of surfactant proteins by AT2 cells. As expected, the transdifferentiation of lipofibroblasts into myofibroblasts is also inhibited by PTHrP [68,87]. PTHrP deficiency or defect in PTH-1 receptor activation attenuates the proliferation and differentiation of AT2 cells, and instead of alveolarization, angiotensin II is produced, which further damages the lung [68,88,89].

An interesting study was conducted to elucidate the novel role of TGF-beta type I receptor tyrosine kinase (ALK5)-mediated TGF-beta signaling, which also regulates the balance between αSMA^+^ myofibroblast and lipofibroblast fate choice and differentiation during lung development [90]. αSMA^+^ cell commitment is regulated by TGF-beta, which acts upstream of the PDGFA/PDGFRα pathway. The study revealed that the Alk5 mutant lung contained a reduced number of αSMA^+^ cells and correspondingly, an increased number of lipofibroblasts. The authors proposed a model whereby ALK5-mediated TGF-beta signaling regulates αSMA^+^ versus lipofibroblast cell differentiation through direct and indirect modulation of target signaling pathways and transcription factors, including PDGFRα, PPARγ, the myogenic transcription factor PRRX1, and the adipogenic zinc finger transcription factor ZFP423.

Taking into consideration the described diversity of lung fibroblasts, data on the contribution of the other subpopulations, except SCMFs, to elastin and collagen synthesis is insufficient [91]. Recent single-cell sequencing analysis clearly identified several lung matrix fibroblast types, but their developmental and/or regenerative role and location remain elusive [46,52,92,93]. Based on transcriptional profiles, matrix fibroblasts are more specialized to form and modulate the ECM during development and repair, while myofibroblasts produce a matrix and also contract and provide tensile strength to the alveolus [46,52].

Quite different data may be acquired based mainly on the molecular-bioinformatics approach for the characterization of the iReF lineages (Table 2). Using an iterative cell type identification strategy, the group of investigators unbiasedly identified the heterogeneity of murine pulmonary populations of epithelial, endothelial, mesenchymal, and immune cells each containing distinct subpopulations [92]. They integrated the scRNAseq analysis of P1 mouse lung with developmental RNA profiles obtained from whole lung tissue. In this study, mesenchymal cell subtypes were largely defined by the expression gradients of cell-selective markers, while cell-specific markers were not readily discerned. The expression of a gene in a cell type was represented by its average expression in all the cells of this type. Pearson’s correlation based on distance and Ward linkage were used by the authors. We will mention here only their results concerning the mesenchyme in order to demonstrate the difference between this approach and the paradigm of compiling the set of subpopulation markers according to lineage-tracing studies (Table 1). They distinguish the following groups of mesenchymal cells: pericytes 1 and 2, matrix fibroblasts 1 and 2, myofibroblasts 1 and 2, smooth muscle cells, and contaminants (Table 2).

The authors themselves note that despite a recent scRNAseq analysis of adult mouse lung that predicted four distinct subtypes of fibroblasts, including lipofibroblasts [52], and though they also found matrix fibroblasts in the neonatal mouse lung, the difference in signature genes defining their subtypes were evident and no clearly defined lipofibroblasts were observed on P1 [92]. In a recent study by Zepp and colleagues utilizing scRNAseq on whole fetal and postnatal mouse lungs (E12.5, E15.5, E17.5, P3, P7, P15, P42), several subpopulations of mesenchymal cells were identified [5]. These were SCMFs (Stc1^+^), progenitor and differentiated airway smooth muscle cells (Acta2^+^), progenitor and differentiated vascular smooth muscle cells (Acta2^+^Pdgfrb^+^), myofibrogenic progenitor cells (Axin2^+^Pdgfrb^+^), MANCs (Axin2^+^PDGFRα^+^) and their progenitors, Wnt2^+^Pdgfra^+^ cell type, and also two proliferative indefinite populations, prolif.1 and prolif.2, and three Pdgfra^+^ cell populations: Pdgfra^+^1, Pdgfra^+^2, Pdgfra^+^3. It is remarkable that in this study, neither lipofibroblasts nor pericytes and populations of Lgr6^+^ and Lgr5^+^ fibroblasts were mentioned. Earlier, Lee and his colleagues showed that mesenchymal Lgr6^+^ cells belong to a population of smooth muscle cells surrounding airway epithelium and promote epithelial progenitor differentiation in the bronchiole via Wnt-Fgf10 cooperation; meanwhile, Lgr5^+^ mesenchymal cells are located in alveolar compartments and are essential for the alveolar differentiation of epithelial progenitors via the activation of Wnt. These are Lgr5^+^ cells that promote the maintenance of self-renewing AT2 cells [59].

The bioinformatics approach was also utilized in the following study, and we would like to elucidate its results as we consider this study to be important. As we already mentioned, these types of studies use another paradigm, which is why some vague matrix fibroblasts expressing the cell surface marker CD106 (Vcam1) were investigated (which, in fact, could be precursors of either myofibroblasts or lipofibroblasts, because they were positive for both Tcf21 and Fn1 on E16.5), ones that originated from the “proliferative mesenchymal progenitor cell” (PMP), as the authors called these cells [93]. In this study, it was not emphasized what kind of markers were used for PMP identification. The authors identified gene networks controlled by glucocorticoid receptor signaling in pulmonary mesenchymal cells during lung sacculation [93]. Glucocorticoid signaling is known to promote the transition of bronchiolar to alveolar cell fate in peripheral epithelial lung progenitors and control the timing of alveolar epithelial cell maturation [94,95,96]. Bridges and colleagues, in their turn, showed that the glucocorticoid receptor enhanced the differentiation of newly defined PMP into matrix fibroblasts, and that the latter played an important role in the synthesis of the ECM and collagen necessary for lung development and survival at birth. This effect was achieved in part by the direct activation of ECM-associated target genes, including Fn1, Col16a4, and Eln, and by modulating VEGF, JAK-STAT, and Wnt signaling. The loss of mesenchymal glucocorticoid receptor signaling blocked fibroblast progenitor differentiation into mature matrix fibroblasts, which, in turn, increased the proliferation of SOX9 alveolar epithelial progenitor cells and inhibited the differentiation of mature AT2 and AT1 cells. Via an integrative analysis, potential glucocorticoid receptor target genes and associated pathways switched on in pulmonary matrix fibroblasts were identified, indicating that “extracellular matrix organization”, “collagen biosynthesis”, and “elastic fiber formation” were highly induced by corticosteroids. Furthermore, Cyr61 (cysteine-rich protein 61), Dpt (dermatopontin that interacts with FN1), Eln, Fn1, Vcam1, and the collagen family Col6a2, Col6a3, and Col14a1 markers were selectively expressed in matrix fibroblasts on E18.5. Their expression was induced by dexamethasone and suppressed by glucocorticoid receptor gene Nr3c1 deletion. Furthermore, ECM genes, including Cyr61, Col6a2, Col6a3, Fn1, and Eln, were shown as direct glucocorticoid receptor targets in matrix fibroblasts, which modulate ECM maturation and function [97].

It has to be pointed out that in a recently created web tool called LGEA [98], used for mapping single-cell gene expression in the developing lung, five mesenchymal cell subtypes are represented: matrix fibroblasts, myofibroblasts, smooth muscle cells, pericytes, and PMP; several markers from the abovementioned studies are specified for these cell subtypes [92]. Nevertheless, the choice of markers is not so obvious. Studies based on single-cell transcriptomic profiling utilize a great amount of multimeric data sets, which are usually impossible to classify using canonical markers. The lung is one of the most sophisticated organs, and morphogenetically active mesenchyme is very heterogenic, making it difficult to characterize cell composition. We can hypothesize that the focus of identifying mesenchymal subpopulations will be shifted to the determination of regulating signaling events in future studies. There is thus a necessity to change the established concept that either one marker (or one set of markers) is exclusively associated with the exact cell type, or that a single cell type controls one corresponding biological process. It is becoming more obvious that one set of genes may be expressed in several cell types, while multiple cell clusters may participate in basic biological processes. Several cell types (or clusters) may transcribe genes associated with a given function. This orchestrated gene expression serves as a response to the shared microenvironmental signals.

## 5. Retinoic Acid Signaling and Alveologenesis

Retinoic acid (RA) plays an important role in the formation of various organs, especially the respiratory system. RA signaling influences lung specification, branching morphogenesis, and alveolarization by regulating the expression of the corresponding target genes. Vitamin A derivatives are crucial for the newly formed septa because they increase the expression of elastin genes and elastin synthesis [99,100,101]. At the end of embryonic development, the maximum amount of retinyl esters is observed in lipofibroblasts, which were shown to produce RA; in the first days after birth, they begin to transform into active forms (all-trans retinoic acid (ATRA) and retinol) through the corresponding metabolic pathways [71,102,103]. Furthermore, the synthesis of RA, RA receptors, and cellular retinol-binding protein (CRABP) mRNA in lipofibroblasts increase during alveolarization [71].

It has been demonstrated that in rodents, ATRA not only induces the formation of alveoli, but it also prevents the inhibition of alveolar formation by glucocorticosteroid hormones and even partially restores it after the cessation of their exposure [104]. Various RA receptors (RARs) modulate the formation of alveoli in the course of their development [99]. The lack of RARα in mice does not alter the number of forming alveoli during perinatal development, but decreases their number after it [104]. In contrast, RARβ signaling inhibits alveolar formation during the perinatal period rather than after [104]. RARγ and RXRα (retinoid X receptor) are required for a complete postnatal alveolar formation in mice [105].

Treatment with RA in a lung recovery model induced alveolar regeneration after partial PNX in aged mice, which lungs no longer regenerate themselves. Retinoic acid was shown to indirectly induce reciprocal PDGF-A signaling, which activates regenerative fibroblasts supporting alveolar epithelial cell differentiation and repair [106].

In light of the results of Massaro and colleagues, McGowan and colleagues, and others suggesting that RA is synthesized by lipofibroblasts during alveologenesis, the recent study by Yun and colleagues, in which RA was shown to be produced by endothelial cells, is intriguing and has created a need to focus on the issue. Yun and colleagues conditionally inactivated the vascular endothelial growth factor (VEGFA) gene selectively in respiratory epithelial cells during postnatal alveolar development, when it is at its maximum expression, and showed that this causes a significant decrease in the formation of alveoli and alveolar capillaries. Furthermore, they demonstrated that the RA synthesized by endothelial cells in response to VEGFA from the epithelium controls elastin production by SMCFs via the induction of their specific fibroblast growth factor FGF-18. The suppression of RA synthesis in neonatal mice, respectively, decreased the expression of FGF-18 and elastin, and thus impaired alveolarization. The simultaneous administration of RA and vitamin A partially restored the impaired vascular and alveolar development induced by VEGFA inhibition. It has to be noted that epithelial VEGFA is also required for the development of a distinct endothelial cell population, which is marked by carbonic anhydrase 4 (Car4). Car4^+^ endothelial cells are specifically eliminated via epithelial VEGFA deletion, which causes an aberrant enlargement of the alveolar space [6]. Consequently, the epithelial inactivation of VEGFA and the impairment of alveologenesis and alveolar capillaries may result from either the absence of Car4^+^ endothelial cells or the suppression of RA synthesis.

## 6. Elastogenesis and Collagens in Alveologenesis

ECM plays an important role in lung development, normal functioning, and repair after injury. The main components of the matrix are elastin, fibronectin, collagens I, III, IV, VI, and XVII, laminins, proteoglycans, and glycoproteins. Many diseases that affect the structure and function of the lung are accompanied by the dysregulation of basement membrane components [107,108,109]. While it is well known that the overexpression of collagen fibers causes lung fibrosis, there is not much information on the role of collagens in alveologenesis. Loscertales and colleagues showed that collagen IV is essential in alveolar lung patterning and proposed a model in which collagen IV regulates epithelial and endothelial components important for alveologenesis, induces (in part through the PDGFRα pathway) interstitial αSMA myofibroblasts to proliferate and migrate to the tips of the septa during early septation, and later contributes to the extension and final maturation of secondary septa [110]. As Col4a1 mRNA is expressed in the interstitium and the tips of alveolar septa in a pattern similar to tropoelastin, the authors suggest that collagen IV regulates elastogenesis specifically in the αSMA^+^ cell population. Moreover, Col4a1 mutants have an increased number of cells with lipid content (AT2 and lipofibroblasts) and abnormal capillary formation. An interesting observation is that collagen VI promotes lung epithelial cell spreading and increases the rate of “wound healing” in response to a scratch injury via phosphoinositide 3-kinase (PI3k) and the cell division control 42 homolog (CDC42) downstream of the interaction with b1 integrins [111].

Among the lung ECM components, collagen fibers (mainly type I and III), which are stiff and strong, provide structural integrity and strength to the lung and are the most important load-bearing element within the alveolar duct and wall, while elastic fibers provide tissue resilience [112]. Degradation of elastic fibers by elastases (e.g., smoking induces the secretion of elastases from inflammatory cells, and mice deficient in macrophage elastase were resistant to tobacco smoking that normally provokes emphysema in wild-type mice, which was shown by [113]) or aging give rise to emphysema. The formation of functional elastin fibers, which give elasticity to the alveoli, plays a crucial role in the process of alveologenesis. Tropoelastin, a soluble monomer of mature elastin, is a spring-like molecule that is extremely flexible and extensible [114]. In this manner, elastin itself possesses remarkable durability and thus the ability to undergo many stretch–recoil cycles while maintaining the structural and functional integrity of elastic tissues throughout life. Elastogenesis is a highly hierarchical multistep process [115]. Elastin and fibrillin-1 (FBN1) represent the building blocks of elastic fibers that compose the elastic fiber core and microfibrils, respectively [116]. The formation of elastic fibers requires several accessory proteins, including fibulin-4, fibulin-5, and the latent TGF-beta binding protein, LTBP-4, which shares structural homology with FBN1 [117,118]. In the study on functional interdependence between fibulin-4 and LTBP-4L (a “long” isoform of LTBP-4) and its impact on matrix deposition and function, Heena Kumra and colleagues, referring to other authors, give the following brief description of elastogenesis [119]: Elastogenesis begins with the secretion of fibronectin and its aggregation into a network, which acts as an organizer for the assembling of FBN1 into microfibrils as well as for the storage of LTBP-4 and fibulin-4 [120,121,122,123]. Tropoelastin undergoes self-aggregation (coacervation) and crosslinking by lysyl oxidase (LOX) and LOX-like 1 (LOXL1), two enzymes that belong to the LOX family, thereby yielding the covalent elastin-specific crosslinks desmosine and isodesmosine [124]. Microaggregates of elastin are formed on the cell surface and then deposited onto microfibrils [125,126]. Fibulin-4 binds to LOX [127,128], and fibulin-5 binds to LOXL1 [129], bringing these enzymes in direct proximity to tropoelastin for the crosslinking of its aggregates [130,131,132]. Fibulin-5 was also suggested to promote the activation of LOXL1 since elastic fibers in fibulin-5-null mice were reported to contain the uncleaved inactive form of LOXL1 [133]. Elastin, which is tethered by fibulin-5, does not directly interact with LTBP-4S (a “short” isoform of LTBP-4) but deposits linearly onto microfibrils through the direct interaction of fibulin-5 with LTBP-4S; this, in turn, also mediates the formation of elastin fibers [118]. Fibulin-4 deposits tropoelastin onto the linearly assembled LTBP-4L molecules that are already bound to microfibrils, thus forming nascent elastic fibers. Furthermore, it serves as a molecular extracellular chaperon for LTBP-4L, inducing its stable conformational and functional change [119]. Though two different groups of scientists [118,119] have drawn similar schemes of LTBP-4 assembly with tropoelastin aggregates via fibulins 4 or 5, respectively, these fibulins had been previously shown to have distinct locations by immunoelectron microscopy. Fibulin-4 labeling was preferentially found in the microfibrils surrounding the elastin cores, while fibulin-5 was present at the interface between elastin cores and microfibrils [134]. Additionally, knockout mice experiments revealed that fibulins 4 and 5 have non-compensatory roles in elastogenesis [135,136]. Thus, despite the notion presented above that this process seems to reveal very similar roles of two congenerous proteins, the questions on why these twin passways exist and how they intersect remain, which may be addressed in future studies.

Tomoyuki Nakamura provided a comprehensive overview of ECM elements, especially short fibulins [137]. Fibulins 3, 4, and 5 are called “short” because of their length; on the other hand, they are grouped into a subfamily based on phylogenetic analysis [138]. In mice, the maximum fibulin-3 protein level is observed in the lungs. Despite this, it was shown to react weakly with tropoelastin, and thus its specific role in the assembly of elastin fibers is unclear [134]. An alternative name for fibulin-4 is epidermal growth factor (EGF) containing fibulin-like extracellular matrix 2 (EFEMP2), although all fibulins contain calcium-binding EGF-like domains. Fibulin-4 mRNA in mice is also most expressed in the lungs [139], but its protein level is much lower than that of fibulin-3 [134]. As mentioned above, fibulin 4 is crucial for elastogenesis, especially for crosslinking elastin molecules; in the lung of mice with its knockout elastic laminae were not formed, and desmosine, a crosslinked amino acid product, was largely diminished [139]. A histological analysis demonstrated that fibulin-4^−/−^ mice did not develop normal elastic fibers. In addition, electron microscopy revealed highly unusual elastin aggregates, as they contained evenly distributed rod-like filaments in contrast to the amorphous appearance of normal elastic fibers [136]. Unlike other short fibulins, fibulin-4 was shown to interact with collagens [134]. The mRNA expression of fibulin-5 (also known as DANCE or EVEC) in the lung of mice is high [140,141,142], and the protein content is significantly higher than that of fibulins 3 and 4 [134]. Nevertheless, the absence of fibulin-4 causes a much more severe perinatal lethal elastic fiber phenotype than the loss of fibulin-5 [130]. As expected, fibulin-5-null mice show overlapping phenotypes with Loxl1-null mice, including emphysematous lungs [129]. Fibulin-5 mRNA expression was induced in the recovery phase after lung injury by hyperoxia and by elastase treatment [143,144]. Data from artery and lung injury models also suggest that fibulin-5 may play some role in the repair of elastic tissues. In fibroblast cell culture, fibulin-5 mRNA expression was greatly induced by TGF-beta and largely abrogated by IL-1β [144,145]. Fibulin-5 interacts directly not only with tropoelastin [130,134,135,146], LTBP-4 [118], and LOXL1 [129], but also with FBN1 and LTBP-2 [118,130,142,147,148], which may contribute to the recruitment of elastin microaggregates onto microfibrils, and with LOXL2 and 4 [132].

The turnover rate of elastic fibers is generally very slow [149], which interferes with recovery from ECM degradation. Moreover, any breakdown of expression, be it genetic or acquired due to external influences, of the above-listed molecules participating in elastin fiber formation leads to defects in elastogenesis, and consequently, to abnormal alveologenesis [150,151]. Tomoyuki Nakamura [137] points out that any approach to elastic fiber repair would need to incorporate fibulins 4 and 5, together with LTBP-4 and LOX, to be successful. Any drug that increases these factors may be of use in preventing the progression of emphysema. In contrast, inhibiting the function of fibulin-4 and/or LOX could be a novel therapeutic strategy for preventing increased collagen deposition in lung fibrosis, as these proteins participate in collagen assembly.

Elastin is expressed in the lung by several types of cells, including pleural mesothelial cells, airway and blood vessel smooth muscle cells, endothelial cells, and interstitial fibroblasts. Due to this, a system of elastic fibers is formed, which ensures an equal transfer of the applied force to all parts of the lung [91]. Numerous studies show that the formation of alveoli is closely related to the formation of elastin fibers in the respiratory region of the lung. During septation, the formation of elastin fibers reaches its peak. Three phases of tropoelastin expression have been observed in the developing lung, with each phase characterized by a distinct expression pattern [50]. In the first phase, on E15.5, the expression of tropoelastin mRNA was confined to mesenchymal cells lining the proximal tubules of the endoderm. During the second phase of expression, at the saccular stage on E18.5, almost all lung mesenchyme was tropoelastin-positive. It was also expressed in the developing vascular walls. In the third phase, on P7–P14, tropoelastin expression was limited to a population of mesenchymal cells located on the tips of the growing alveolar septa (and to developing cells of the vascular and bronchial walls). Mesenchymal tropoelastin-positive cells detected in the third phase of postnatal expression were specifically and completely absent in the lung of PDGF-A^−/−^ mice, while tropoelastin expression in bronchial and blood vessel walls was unaffected. The impairment of postnatal alveologenesis in PDGF-A^−/−^ mice was suggested to be due to a prenatal block in the distal distribution of PDGFRα^+^ cells [50]. In the absence of a PDGF-A signal, PDGFRα^+^ cells are unable to multiply and spread. Thus, the depletion of myofibroblasts in PDGF-A-null mice results in a lack of elastic fibers in the alveolar walls and incomplete alveolarization [27,50]. Similar effects were observed in Hoxa5^−/−^ murine lung, in which the subset of the mesenchymal progenitors of alveolar αSMA^+^ myofibroblasts expressing Pdgfrα failed to spread into the distal fetal lung [152]. They were trapped in the parenchyma surrounding future alveoli, where they produced abnormal elastic fibers. Elastic fibers were disorganized and aberrantly distributed within the pulmonary tissue on P0 and P5, and alveologenesis was impaired. From P15 onwards, the fibers appeared more abundant, disorganized, and fragmented. Northern blot analyses of tropoelastin expression failed to reveal differences in tropoelastin transcript levels between wild-type and Hoxa5^−/−^ lungs. Later, it was shown that Hox5 genes are required in elastin network formation by regulating the adherence of the alveolar fibroblasts to fibronectin, which is disrupted because of the loss of the integrin heterodimer Itga5b1 in mutant fibroblasts [153]. In Hoxa5 mutants, the activated macrophages were abundantly found in the lung as early as birth, and most of them expressed matrix metalloproteinase 12 (MMP12). This recruitment coincided with the abnormal elastin deposition by the mispositioned alveolar myofibroblasts, supporting the notion that elastin fragments are chemotactic for monocytes [154]. Furthermore, recent studies showed that the absence of Pdgfra in Gli1^+^ SCMFs disrupted the expression of elastogenic genes, including members of the LOX, fibronectin, and fibulin families, while the expression of the EGF family members was increased, and Tgfb1 was suppressed in the lung fibroblasts of the studied mice. Similar results were found in lung samples from a person with BPD. Blocking PDGF-A signaling in vitro had the same effect. In addition, the effect was reversible by inhibiting EGF or activating TGF-beta signaling. These data demonstrate the role of PDGFA/PDGFRα in the control of elastogenic gene expression through the secondary level of signaling networks consisting of EGF and TGF-beta [80]. Finally, the growth factor FGF-18 was also shown to affect myofibroblast differentiation by stimulating the expression of tropoelastin, LOX, and fibulins 1 and 5 during secondary septation [51].

Some methods that suppress alveolarization, such as prolonged hyperoxia (BPD modeling), also suppress septation and elastin expression in the alveolar wall [155,156,157,158]. Elastin fiber assembly impairment in newborn rats due to copper deficiency [159] or the administration of beta-Aminopropionitrile (beta APN) [160], which interferes with the synthesis of elastin and collagen by inhibiting LOX, results in the impairment of alveolar septa formation and the simplification of the alveoli by the type of emphysema. In contrast to PDGF-A^−/−^ mice, in which the absence of alveolar elastin synthesis and the abnormal structure of the alveoli are detected after P4, the inactivation of the elastin gene in Eln^−/−^ mice leads to a delay in the perinatal development of the terminal airway branches. At birth, these mice have enlarged cavities in the lung that resemble emphysema [161]. These differences indicate that, in addition to its role in the formation of the structure of alveoli, elastin is important for the terminal branching of the airways in lung development. This also supports the idea that elastin is expressed by airway smooth muscle cells, which enwrap the branching tips and the stalk of the primordial airways and physically support branching morphogenesis [162,163].

## 7. Alpha-Smooth Muscle Actin and Secondary Septa Eruption

**Figure 3:**
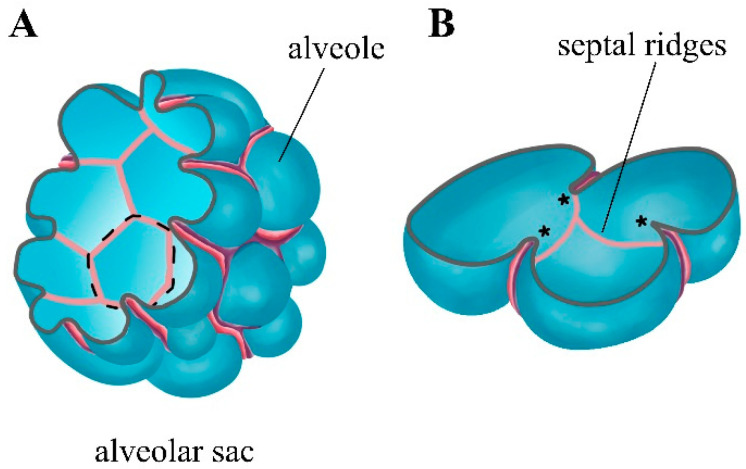
Cross-section of the alveola with a view of the secondary septa. αSMA fibers, marked in pink, and closely related elastin matrices, marked in purple, are expressed in an organized network, mimicking a “fishnet” pattern in the alveolar region. The alveolar epithelium is marked in blue. The black dotted line indicates the entrance alveolar ring (AER) (**A**). Septal ridges in 2D look like an assembly of αSMA fibers on the tips of secondary septa (asterisks) (**B**). The gray line imitates the cutting line. Redrawn from [39].

How researchers identify the subpopulations of myofibroblasts involved in the formation of secondary septa, and the process of septa expansion itself, are important for understanding the driving forces of alveologenesis. In 2016, Branchfield and colleagues, using a 3D modeling of alveologenesis at different stages, quite clearly showed that septal ridges, some in the form of circular rims at the alveolar entrance ring (AER), were all lined by αSMA fibers on P7 (Figure 3) [39].

Previously, PDGFRα^+^ myofibroblasts involved in septal formation were reported to be located at the AER [47]. However, there were no clearly visible gaps in these αSMA^+^ rings. It is probable that myofibroblasts had elongated cytoplasmic protrusions and tight or overlapping junctions that form a continuous loop around the entrance ring of the alveoli. In addition, these rings were interconnected in the αSMA network, which resembled a “fishing net”. Taken together, the data demonstrated in this work suggest that a more accurate representation of growing septa is the elongated ridges (“septal ridges”) in 3D that appear to rise from the base of the alveolar bowl in order to subdivide large alveoli into smaller ones. Septal ridges have a scaffold of αSMA^+^ myofibroblasts and associated elastin fibers. As this pattern suggests the presence of mesh strands, which underlie future alveolar ridges, the authors hypothesized that the coordinated contraction of these strands comprising αSMA^+^ myofibroblast network “filaments” would lift the ridges into the lumen and/or provide a tension force in order to withstand air pressure against the alveolar surface. In this or that way (or both), the alveolar surface covered with filamentous myofibroblasts will form the AER [39]. McGowan and colleagues noted that the accumulation of αSMA^+^PDGFRα^+^ myofibroblasts at the AER occurs earlier after birth than the accumulation of elastin; most of the circumference of the AER was occupied by αSMA rather than elastic fibers on P4. However, this discrepancy disappeared by P8, and by P12, the αSMA occupied a smaller part of the circumference than on P8. Since mechanical tension in the AER is required to prevent alveolar collapse during expiration, αSMA may have a more important mechanical function on P4 than on P12, when there are more elastic fibers in the AER. Thus, αSMA may play a role in stabilizing the AER during expiration until elastic fibers begin to accumulate [47]. Since 3D analysis revealed secondary septa to be in the form of rings containing elastin and collagen, it is believed that the relative stiffness of the AER border determines the orientation of individual alveolar components of the gas exchange surface that form the alveoli, and serves to delineate and stabilize the primordium of the alveolar epithelium. Thin epithelium lining lateral alveolar duct buds may extrude or “pop-out” from the duct lumen through rings more similar to soap bubbles or chewing gum bubbles. It is obvious that the property of the lung surfactant to decrease tension may facilitate this process as well as stabilize the alveolar epithelium and prevent collapse after the budding of alveoli [164]. In this model of alveologenesis, preference is given to the extrusion/squeezing of the epithelium through the orifice rather than the commonly accepted notion of alveolar formation by the raising of the finger-shaped alveolar septa, which compartmentalize the saccula, a process that requires the contractile properties of specialized alveolar myofibroblasts [164]. Interestingly, compared to the normal lung, impaired lung development causes the increase and disorganization of αSMA and elastin fibers. In pathologic conditions, the ordered structure of the “fishnet” turns into a “cheesecloth”, which is ineffective in driving the differential rise of septal ridges from septal walls [39].

Understanding the mechanisms of septa thinning is also important since septa thickening on P1–P14 inhibits the formation of alveolar capillaries, such as when the connective tissue growth factor (CTGF) is overexpressed, which ultimately leads to the increased differentiation of myofibroblasts and the disorganized deposition of elastic fibers in the alveolar septa, thus causing fibrosis [165]. In a lung regeneration model, it was shown that αSMA expression could be reactivated during lung regrowth and regeneration. FGF and PPAR-γ signaling are required for the induction of αSMA in PDGFRα-positive myofibroblast progenitors during compensatory lung growth [46,48,49].

The data suggest that instead of determining the fate of differentiated myofibroblasts, αSMA expression may represent a contractile phenotype that PDGFRα^+^ cells may temporarily adopt during septation, stress, or regeneration. As noted above, during normal lung development, the formation of secondary septa is associated with the transient presence of αSMA-positive interstitial myofibroblasts. After P15, αSMA expression is significantly reduced, and SCMFs are expected to be eliminated. Recently, in contrast to previous data showing that Pdgfra^+^ cells equally give rise to myofibroblasts and lipofibroblasts, ~95% of Pdgfra^+^ lineage tracing cells were shown to be myofibroblasts via PdgfrartTA knockin mice and the CRISPR/Cas technique [61]. The genetic ablation of Pdgfra^+^ cells led to a simplification of the alveoli, demonstrating that these cells are required for the formation of septa, as was already mentioned. Data from numerous studies demonstrated that in pathological conditions such as BPD and a neonatal hyperoxia mouse model of BPD, smooth muscle markers continue to be detected in the alveolar region, indicating the persistence of myofibroblasts. In the model of bleomycin-induced pulmonary fibrosis in adults, the number of Pdgfra^+^ lineage tracing cells increased; therefore, they contribute to pathological myofibroblasts. In contrast, in the BPD model of neonatal hyperoxia, the number of Pdgfra^+^ lineage tracing cells decreased. These data revealed the complexity of the behavior of Pdgfra^+^ cells [61]. It is noteworthy that lineage tracing Pdgfra^+^ cells survive until adulthood (analyzed on P40) [61]. Thus, the expression of αSMA is apparently a transitory characteristic of secondary septa fibroblasts. It is possible that they adapt and perform temporal functions responding to spatial and short-term signals to promote alveolarization. The characterization of the mechanisms that control the emergence and attenuation of αSMA will probably make it possible to understand how the fibroblast niche changes and myofibroblasts arise, as well as the influence of microenvironmental changes on their generation.

## 8. Remodeling of the Cell Cytoskeleton Is Necessary for Alveologenesis

Alveolar epithelial cells, myofibroblasts, and endothelial cells participate in coordinated morphogenesis during alveolar formation. During alveologenesis, the shape of AT1 cells changes, and they form epithelial folds (secondary septa). Myofibroblasts exert a mechanical strain to protrude into the folds and the vasculature in the forming septa remodels. However, the understanding of the mechanisms of alveologenesis remains incomplete. Signaling pathways that control the actomyosin cytoskeleton play a crucial role in cell migration and interaction during the formation of secondary septa. It was recently discovered that planar cell polarity (PCP) is not limited to tissue polarity and plays a distinct role in tissue patterning. The Wnt5a–Ror2–Vangl2 pathway controls the changes in the cytoskeleton of epithelial cells and myofibroblasts, as well as imparts cellular properties required for alveologenesis [166]. PCP signaling has frequently been described to control the collective cellular behavior of epithelial sheets. PCP is a non-canonical Wnt pathway, also called tissue polarity, referring to the polarized organization of cells in the epithelium plane. This concept was primarily formed on the basis of the studies of the Drosophila wings and eyes epithelium [167,168]. PCP components are asymmetrically distributed within individual epithelial cells, which acts as a unique signaling mechanism. PCP protein complexes are capable of generating molecular asymmetry within cells along the axis of the entire tissue, which is then translated into actin polarization and microtubule cytoskeleton dynamics. PCP is an important regulator of developmental processes, homeostasis, and diseases of the respiratory system. It acts in the airway epithelium to establish and maintain the orientation of the respiratory cilia along the airway axis for directed mucociliary clearance; it also regulates the formation of the pulmonary vasculature. In adult tissues, PCP dysfunction is associated with a variety of chronic lung diseases [169].

Wnt5a is a known ligand for the PCP signaling pathway that modulates this pathway. It is an important regulator of stem cell renewal, cell migration, cell polarity, and inflammatory responses. Wnt5a is expressed in both epithelial and mesenchymal compartments at embryonic stages, and mainly in fibroblasts and endothelial cells in the adult lung [60,170]. The dysregulation of Wnt5a has also been observed in lung diseases [171,172]. The inactivation of Wnt5a leads to the impairment of lung development. The absence of Wnt5a in the saccular phase blocks the expansion of the distal airways and attenuates the differentiation of endothelial cells, AT1 cells, and myofibroblasts. The postnatal inactivation of Wnt5a impairs alveologenesis, and the lung phenotype resembles human BPD. Hypoalveolarization has been observed in the mutant lung, but endothelial and epithelial differentiation was not affected. The inactivation of Wnt5a at the stage of alveologenesis disrupts the differentiation and migration of myofibroblasts, which has also been confirmed in vitro and has caused a decrease in the expression of genes enriched in myofibroblasts. The conditional inactivation of Wnt5a receptors Ror1 and Ror2 in alveolar myofibroblasts reproduces the Wnt5a-deficient lung phenotype, demonstrating that myofibroblast defects are the main cause of pulmonary alveologenesis arrest after Wnt5a inactivation. In addition, Wnt5a is reduced in human BPD lung samples, indicating the clinical significance and potential role of Wnt5a in the pathogenesis of BPD [173].

Previously, it was shown that the PCP genes Celsr1 and Vangl2 are required for normal branching morphogenesis. The lungs of two mouse mutant strains, Celsr1 crash (Celsr1^Crsh^) and Vangl2 Looptail (Vangl2^Lp^), demonstrated thickened interstitial mesenchyme, defective saccular formation, and a disturbance of the integrity of the epithelium and the arrangement of the cytoskeleton. It should be noted that homozygous Vangl2^Lp/Lp^ mice die after birth; therefore, heterozygotes are investigated via the inactivation of a single copy of Vangl2. The activation of the Rho signaling pathway in mutant embryos can partially reduce the branching defect. The authors note that these data are consistent with Rho-kinase being a downstream effector of the PCP signaling pathway in lung development. The data also suggest that defective cellular organization in Celsr1^Crsh^ and Vangl2^Lp^ mutants is a result of impaired Rho-kinase function that likely leads to defects in the cytoskeleton, thus disrupting tissue structure [174]. In the postnatal lung, the PCP signaling pathway also plays a significant role. A strong increase in air space and impaired adult lung function were observed in Vangl2^Lp^ mice after birth. In the lungs of Vangl2^Lp^ mice, an altered distribution of actin microfilaments, the abnormal regulation of the actin-modifying protein cofilin, and the impairment of alveolar epithelial cell migration were found. Furthermore, altered macrophage population, abnormal elastin deposition, and elevated levels of the elastin-modifying MMP12 were observed, which are similar to the markers of emphysema. In addition, cytoskeleton abnormalities were found in AT1 cells lacking Vangl2, preventing their folding to form secondary septa. Vangl2 also regulates cytoskeleton and myofibroblast migration. Vangl2-deficient interstitial fibroblasts/myofibroblasts have demonstrated a disorganized actomyosin cytoskeleton and migration defects [166]. Vangl2 expression was inhibited in the lungs of patients with emphysema. In addition, its polymorphism has been associated with the negative effects of smoking on lung function in humans. These data show the novel and important role of the PCP pathway in the homeostasis of the adult lung and its recovery after injury [175].

The role of Vangl2 has also been shown in mechanosignaling. Vangl2 modulates cellular mechanics, including focal adhesion complexes, actomyosin organization, actomyosin-mediated contractility, and traction force generation via RhoA signaling. The impairments of these processes in Vangl2^Lp^ mice lead to a deficiency of the mechanoregulator YAP. The inactivation of Vangl2 is associated with a decrease in nuclear (active) YAP and an increase in cytoplasmic (inactive) phospho-YAP in the lung epithelium [176]. In utero, YAP and its nucleocytoplasmic shuttling are known to be crucial for lung branching morphogenesis [177,178], while TAZ deletion causes abnormalities in postnatal alveologenesis, leading to an emphysema-like phenotype in adult mice [179]. Previously, Park and colleagues found that YAP/TAZ transcription coactivators could act as downstream effectors of the PCP pathway, mediating cell migration through Frizzled and ROR receptors [180]. YAP provides the readout of mechanosignal activity [181,182], while Vangl2 is suggested to play an active role in mechanosignal transmission [176].

Recently, the role of PCP in the Wnt5a–Ror2–Vangl2 axis has been investigated. Vangl2 is required both in the epithelium and in the mesenchyme of the lung for the formation of alveoli. The lung lacking epithelial Vangl2 consisted of enlarged saccules and could not form alveoli on P7. The inactivation of Vangl2 in the lung interstitial fibroblasts/myofibroblasts disrupted alveolar formation, resembling the loss of epithelial Vangl2. Interestingly, the authors found no apparent asymmetric localization of Vangl2 in AT1 and AT2 cells or myofibroblasts. In mice with Ror2 inactivation in the lung epithelium or interstitial fibroblasts/myofibroblasts, alveolar defects mimic the phenotypes caused by Vangl2 inactivation. Mesenchymal Wnt5a was found to trigger a Ror2/Vangl2-mediated cascade in both the lung epithelium and the mesenchyme [166]. The loss of PCP signaling in alveolar epithelial cells impaired their ability to signal PDGF to myofibroblasts in the mesenchyme. Alveolar epithelial cells lacking Vangl2 could not present the PDGF ligand to mesenchymal myofibroblasts. Pathways regulating vesicular transport and release of the PDGFA ligand were disrupted in the lungs of mutant mice. The loss of Vangl1/2 in AT1 cells reduced their ability to form new alveoli after lung injury. In patients with COPD/emphysema, Wnt5a and Vangl2 expression levels are reduced, indicating an association between PCP signaling and alveolar regeneration [166]. This result is consistent with data on the suppression of Vangl2 expression in the lungs of patients with emphysema [175]. The positive feedback loop between Wnt5a and PDGF promotes fibroblast/myofibroblast proliferation. Subsequently, the orchestrated movement of AT1 cells and myofibroblasts is regulated by PCP signaling, including the reshaping of AT1 cells and myofibroblast migration. As a result, they approach each other and undergo coordinated morphogenesis with the formation of secondary septa and alveoli. The active role of AT1 cells in the secondary septa formation contrasts with the existing notion of their passive role in this process that is thought to be mainly controlled by myofibroblasts. In addition, it has recently been shown that AT1 cells serve as a signaling node and a source of ligands for myofibroblasts of secondary septa in the developing alveolus, and that this function is evolutionarily conserved [5].

Thus, the development of secondary septa depends on coordinated changes in the shape of both alveolar epithelial cells and myofibroblasts. A key element in this process is the alteration of the actomyosin cytoskeleton. Moreover, the actomyosin contractility is a central hub coordinating mechanosensing and mechanotransduction responses.

## 9. Lung Regrowth and Neo-Alveolarization

Early assumptions concerning lung regeneration involved the idea that lung developmental programs are reactivated during regeneration, similar to limb regeneration in urodeles [183]. However, Kho and colleagues later theorized that post-PNX lung regeneration recapitulates later stages of lung development, when alveoli are formed, rather than earlier stages [184], because of the observed transcriptomic similarities between these processes and more recent data obtained by other scientists [42]. We contemplate that the group headed by Voswinckel was willing to test this theory since in one of their papers, they described the results of a study on gene expression in both newborn mice undergoing the physiological steps of alveolarization and in adult 10-week-old animals undergoing compensatory lung growth after the resectioning of the left lung [185]. So far, it has become obvious that the truth is somewhere in between since even Wolff and his colleagues found little overlap in their comparative study, which reflects either the importance of a few key regulators or the wide independence of the processes. In our review, we would like to present the available data on the mechanisms of lung regeneration after unilateral PNX in order to elucidate relevant candidate drivers for alveolarization, as knowledge of alveolarization and neo-alveolarization taken together can allow for the identification of new target candidates that can be used for the development of therapeutic approaches in the treatment of pulmonary structural diseases.

In many mammalian species, the removal of one lung results in the rapid compensatory growth of the remaining lung almost to the extent of the intact lung. In humans who had undergone lung resectioning, compensatory lung growth was suggested to play a significant role in children less than 5 years of age, while in adults, functional adaptation was predominant [186,187]. In patients younger than 4 years, alveolar multiplication may contribute more to the compensation of lung capacity after lobectomy than an increase in alveolar size; however, after 4 years, compensation for pulmonary function is thought to occur mainly due to the overinflation of the alveoli. Moreover, the overinflation of the remaining lung does not result in symptomatic emphysema after lobectomy in children, even after the age of 4 years, while some adults obtain restrictive as well as obstructive dysfunction [187]. Interestingly, there is at least one piece of evidence that compensatory growth may occur in adults. A 33-year-old woman who had undergone a right-side PNX had an increase in the number of alveoli 15 years later, but the alveoli in the growing lung were shallower [188]. Regarding animals, Voswinckel et al. demonstrated the restoration of normal lung volume in mice, accompanied by the complete regeneration of alveolar gas exchange surface area (and total alveolar septal volume) [189]. At the same time, the number of new alveoli added to the residual lung compensated only for 49% of the alveoli lost during left lung resectioning, while a 100% regeneration of the organ volume was observed [190]. Thus, compensatory lung growth in this mouse model is achieved by both adding new alveoli and increasing the size of already existing alveoli. Notably, 74% of newly formed alveoli were already present on day 6 after PNX in mice, indicating the rapid induction of new alveoli formation after lung resectioning [190]. Various data suggest that the formation of new alveoli is primarily initiated in the subpleural regions of the lung. It was shown that proliferation, which was identified by the autoradiography of injected tritiated thymidine, peaked on day 2 after PNX in mesothelial cells, while the proliferation of alveolar tissue peaked on day 4. Consequently, the labeling of pleural mesothelial cells peaked before the labeling of other types of cells [191]. In addition, the continuous labeling of proliferating cells with bromodeoxyuridine (BrdU) for 10 days revealed areas of BrdU-positive thickened septa in the subpleural regions [189]. Post-PNX lung growth involves more than simply adding new cells through a simple process of cell proliferation and maturation. The transformation of the alveolar outgrowth in the intact part of the lung after PNX, as in normal development, involves the construction of a complex alveolar microarchitecture [189,190], with stringent requirements for the structure of the secondary septa and the gas exchange capacity. It was demonstrated that after PNX, DNA synthesis and cell proliferation are rapidly induced, the number of AT2 cells as well as the number of other major types of resident cells required for the formation of new alveolar septa increase, and the total capillary and alveolar epithelial surface also increases [189,191,192,193,194]. This is preceded by the induction of transcription factors and the activation of signaling pathways involved in lung development and repair [195,196]. During both realveolarization and alveologenesis, various isoforms of (pro-)collagen molecules, elastin, and the elastin-associated protein FBN1 are upregulated [185]. Accordingly, the formation of new alveoli includes a triad of spatially different processes, namely the formation of the septum (including matrix and tissue remodeling), the proliferation of the epithelium, and capillary angiogenesis, which are associated with the overall effect of pleural and remaining lung deformation after PNX [197]. It is worth noting that the processes of restoring the volume and the number of gas exchange units are essentially induced by transpulmonary pressure (mechanical stress and stretch), which was shown in models when, for example, the possibility of the right lung to expand after a left-side PNX is completely eliminated [198,199]. Thus, the expression of lung fibroblast chymotrypsin-like elastase 1 (Cela1), which is important for elastin remodeling during lung development and regeneration, and its binding to areas of lung elastin remodeling were shown to be induced by stretch [200]. YAP is considered to be a transcription coactivator, which is known to be a key nuclear effector of mechanical tension [201], as mentioned before. It was shown to have a crucial role in regulating alveolar regeneration by the induction of nuclear YAP expression in AT2 cells and their proliferation in response to increased mechanical tension after PNX [199]. Upstream of YAP, small Rho GTPase Cdc42-mediated F-actin remodeling acts as an activator of both JNK and p38 MAPK signaling for the activation of YAP [199]. In addition, the deformation of the pleura after PNX leads to an epithelial–mesenchymal transfer (EMT) (or mesothelial–mesenchymal transfer of the pleura), a process in which cell–cell contacts are disrupted and the shape of cells changes [202]. Between 1 and 3 days after PNX, the number of cells expressing αSMA in the pleura significantly increases, as shown by Ysasi et al. Transient cells acquire a mesenchymal migratory phenotype and migrate to the subpleural alveoli. Similar to developmental processes, these migrating myofibroblasts, such as SCMFs, are involved in the redistribution of the alveolar duct. PDGFRα-expressing iReFs play a significant role not only in alveolarization but also in alveolar regeneration [27,47,48,49]. Green and colleagues revealed the two phenotypically dynamic and functionally diverse groups of interstitial PDGFRα-expressing fibroblasts during lung regeneration and realveolarization after partial PNX. They suggest that the majority of PDGFRα^+^CD34^+^ iReFs are transitional lipofibroblasts and that their depletion 5 days after surgery indicates their transformation into CD29-, αSMA-expressing myofibroblasts. Furthermore, the authors speculate that FGFR2 signaling is important for myo-iReF but not matrix-iReF differentiation, whereas PDGFRα signaling is necessary for the activation of matrix-iReFs in the process of realveolarization [46]. After PNX, an increase in the number of AT2 cells precedes the multiplication of AT1 cells. The proliferation of AT2 cells and their differentiation into AT1 cells provides a mechanism for the epithelialization of the alveolar surface required for new alveoli [203]. On day 7 after PNX, AT2 cells were compared with a nonsurgical control, and a notable expression of genes associated with inflammation (Ccl2, Cxcl2, Ifng) as well as genes associated with epithelial growth (Ereg, Lep) was observed. AT2 cells appear to recruit blood-borne monocytes into the lungs [203,204]. Recently, CD115^+^ and CCR2^+^ monocytes and M2-like macrophages were shown to move to the lung via the CCL2–CCR2 chemokine axis and accumulate there during the peak of AT2 cell proliferation, modulating the proliferation and differentiation of these cells. The primary source of CCL2 signaling appeared to be AT2 cells. The authors also provided evidence that group 2 innate lymphoid cells are the source of proinflammatory cytokine IL-13, which is known to modulate the expression of MMPs [205], discussed below, and promotes lung regeneration [204].

Although myofibroblasts, AT2 cells, and blood-borne monocytes contribute to the growth and patterning of alveolar septa and epithelium, it is less clear how these cells may influence the formation of new capillaries within the septa. To date, the available studies are mainly devoted to research on the effect of endothelium on alveolarization, and not vice versa. Thus, the idea that considers alveolarization as the driving force of vascularization is poorly recognized. The epithelial expression of Vegfa has only recently been demonstrated to be responsible for the establishment of the Car4^+^ endothelial cell population critical for alveologenesis [6]. It is established that sprouting and intussusceptive angiogenesis are pivotal processes in adult lung alveolarization after PNX, especially in the subpleural areas, which provide a mechanism for the rapid expansion of the vascular network [206,207]. The blood-borne population of CD34^+^ endothelial progenitor cells characterized by increased proliferative activity and an amplified transcriptional signature is incorporated into the vascular lining of the regenerating lung and makes an important contribution to pulmonary angiogenesis [208]. The rapid augmentation of gas exchange units suggests an extremely efficient alveolar capillary angiogenesis process. Considering the fact that interalveolar septa make no sense without capillaries, the stimulation of angiogenesis is an attractive tool for influencing the outcome of regeneration. Paracrine erythropoietin (EPO) signaling was demonstrated to be involved in normal lung growth and regrowth after PNX, with concurrent activation; however, this occurs with the differentiated processing of the erythropoietin receptor [209]. When administered the nebulization of recombinant human EPO-containing nanoparticles via a permanent tracheal stoma weekly for 16 weeks, paracrine EPO signaling in the lung selectively enhanced alveolar angiogenesis during compensatory lung growth after PNX in dogs [210].

Endothelial cells, in turn, play an important role in stimulating AT2 proliferation during neo-alveolarization. VEGF accelerates compensatory lung growth after PNX in mice by increasing the number of alveolar units [211]. These changes can be mediated by both VEGFR2 and EGF-dependent mechanisms [212,213] and a paracrine mechanism through the upregulation of epithelial cell mitogen HB-EGF [214]. According to Ding et al., the activation of VEGFR2 and FGFR1 induced the capillary endothelial cells of the remaining lung lobes to produce the angiocrine membrane-type MMP14 (also known as membrane-type 1 MMP, MT1-MMP), which is critical for ECM remodeling. MMP14, in turn, activated the EGF receptor (EGFR) by unmasking cryptic EGF-like ectodomains, and this stimulated the proliferation of epithelial progenitor cells [212]. Stromal cell-derived factor 1 (SDF-1) from platelets also deployed MMP14 via receptors of chemokines CXCR4 and CXCR7 on pulmonary capillary endothelial cells. This stimulated alveolar epithelial cell expansion and neo-alveolarization [215]. Another factor that regulates the expression of MMP14 is the transcription factor FOXF1, which is also expressed by endothelial cells and acts by effecting the MMP14 tissue inhibitor, ECM remodeling gene Timp3 [216]. In accordance with the abovementioned studies, it is not surprising that MMP14 plays a role in the formation of the capillary network and distal airspace branching during alveolarization. The alveolar surface area of mice lacking MMP14 decreased by 40% at 1 month of age; the mice also had alveoli with abnormal ultrastructural appearance and enlarged airspaces [217,218]. It is likely that other MMPs also play a role in neo-alveolarization after PNX, as a normal expression of different MMPs and their endogenous inhibitors TIMPs in the lung is tightly regulated, showing upregulation in early lung development, remodeling in response to tissue injury, and host defense against pathogens [219]. However, this area remains poorly investigated. It should be noted that endothelial cells also express YAP1, which is important for compensatory lung growth and vascular and alveolar morphogenesis after unilateral PNX since its knockout inhibits these processes [220]. Notably, defective alveologenesis is observed in normal lung development in the absence of expression of YAP and TAZ in pericytes, specialized mesenchymal cells that help maintain the integrity of the vascular wall [221].

As we have already elucidated, previous studies of lung growth after PNX in mice have identified regenerative hotspots in the subpleural alveolar ducts. The single-cell transcriptional profiling of these hotspots showed that several cell clusters were the most transcriptionally active in the regenerative alveolar duct. Cluster 1 included fibroblasts, CD34^+^ cells, and AT2 cells; in cluster 2, cells of probably a transitional type were grouped as well as monocytes and alveolar macrophages; cluster 3 contained CD31^+^ endothelial cells. The authors identified six cell clusters in total [222]. Data from this study indicate that cluster 1 is comprised of highly active cells that regulate multiple functions during lung repair and regeneration. The authors make the following valuable conclusions regarding bulk data analysis and the approach to identifying cell subpopulations. There are several cell classifications: classification based on the properties of cells, which helps to determine cell functions; classification based on cell morphotypes acquired from microscopic data, which demonstrates the size, shape, and ultrastructural features of cells; cell classification based on molecular phenotype connected to the expression of molecules on the cell surface; cell classification based on genetic signature by gene expression profiling. The latter classification came into general use owing to the development of the single-cell transcription profiling approach. However, progress with this approach is limited to the large, multidimensional datasets that are often impossible to juxtapose with classifications using “traditional” markers. Moreover, there are problems associated with normalizing different datasets. Conclusions based on aggregate data have to be viewed as hypotheses or preliminary data predictions that need to be verified in future research [222].

To conclude, we have to note that the regeneration of the lung and the alveoli in particular is a complex process that involves many cell types and utilizes cell proliferation and differentiation, angiogenesis, and parenchymal remodeling. Data from studies on lung recovery after PNX indicate important differences between developmental morphogenesis and compensatory lung growth, although features of bulk alveolarization can be detected in the course of compensatory adult lung growth. In the case of lung regeneration, the timing and patterning of regrowth are less clear because it utilizes not all but certain signaling pathways of those activated during lung ontogenesis. The functional result of the regeneration is the regrowth of a lung capable of maintaining gas exchange performance.

## 10. Conclusions

The main mechanisms that contribute to organizing the complex process of lung septation remain mostly understudied. The definition of the diversity of pulmonary cell types, their molecular signatures as well as interdependent signaling events, cell–cell and matrix–cell interactions, and the influence of mechanical signals both in development and during regeneration contribute to understanding alveolarization. However, to ensure that new therapeutic concepts can be implemented in the treatment of pulmonary diseases in humans, the validation of the identified candidates and pathways in human material has to be considered.

Unlike the notion of the well-defined role of septal epithelial cells in the alveolar niche, which are not so plastic, the assumption that being a functional stage rather than a defined lineage probably allows the fibroblast to quickly adapt to dynamically changing environmental signals (paracrine signals, mechanical tension, stress and loss of epithelial cells, and inflammation), which seems to be quite reasonable. In our overview, we tried to classify the heterogeneous pulmonary mesenchyme with the definition of the roles of different types of fibroblasts in septa formation during alveologenesis. Signal cues that make fibroblasts follow or change their developmental and homeostasis programs are of particular interest. For example, understanding the nature and development of lipogenic versus myogenic fibroblast phenotypes might help to develop new therapeutic strategies for pulmonary diseases. Despite a large amount of data, there is still no established view on the grouping of iReFs, and researchers do this in different ways [26,46,93]. Moreover, many markers of these populations may overlap. In addition, the rationale for the choice of markers for lineage tracing becomes obscure in light of the heterogeneity and fuzziness of the peak expression values obtained by single-cell sequencing for previously established subpopulations. The structure of the cell population that was likely determined with the use of classical experiments on lineage tracing based on marker identification has yet to be correlated with the new data revealing the dynamics of cell–cell interactions and the activation of signaling pathways. The question remains whether septal fibroblasts are initially determined and/or have plasticity, and if they can change their paths during alveologenesis depending on the incoming signals, as it was shown, for example, in the processes occurring during realveolarization after unilateral PNX. What also remains to be studied is the question of which subpopulation of fibroblasts is eliminated from septa and which mesenchymal cells remain there during continued alveologenesis and adulthood. It was demonstrated that ECM, composed of various molecules of collagen, fibulins, tropoelastin, fibronectin, and others, plays a crucial role in all stages of preparation for secondary septation during its course, and in the recovery of the lung after injury. The accumulated knowledge suggests that ECM not only fills the space between cells but is an active participant in the processes of morphogenesis and regeneration since it affects the proliferation, migration, differentiation, and functioning of pulmonary epithelial and stromal cells. As elastogenesis plays a crucial role in lung morphogenesis, the modulation of this process is proposed as a prospective target for therapeutics. The remodeling of the cytoskeleton plays an important role in alveologenesis and realveolarization since during morphogenesis and regrowth, the spatial organization of cells and structures changes, and tension and mechanical stress arise, which trigger various signaling pathways. PCP signaling was shown to have novel functions in tissue patterning. The Wnt5a-Ror2-Vangl2 cascade controls changes in the cytoskeleton of lung cells, which is critical for the formation of secondary septa. Regeneration after PNX in animals is performed via compensatory lung growth, which makes it possible to more clearly realize the mechanisms and nature of alveolar septal growth. The formation of new alveoli during the regeneration of the remaining lung includes the development of septal tissue, cell proliferation, and capillary angiogenesis that are consolidated by the overall influence of pleural deformation. Studies of lung recovery after PNX indicate important differences between morphogenesis and compensatory lung growth; however, stringent requirements for the structure of the septa and its gas exchange characteristics remain in both cases. Mapping the molecular signatures of the interactions between alveolar mesenchymal and epithelial cells will most likely provide further insights into the nature of alveolar septation.

## Figures and Tables

**Table 1:** Commonly used markers to distinguish different populations of iReFs during the alveolarization phase and perialveolarization period.

iReFs Type	Suggested Markers for the Corresponding iReF Type
Myofibroblast	TBX4 [12]
	ACTA2 [27]
	PDGFRA [27,47,48,49]
	FGF18 [37]
	ELN [50,51]
Matrix fibroblast	COL13A1, COL14A1 [52]
	CD34 [46]
	PDGFRA [46]
Lipofibroblast	THY [53]
	FGF10 [18]
	TCF21 [54]
	PLIN2 (ADRP) [55]
	Leptin [56]
	PPARγ [57]
	PDGFRA [46,58]
Alveolar niche cell	AXIN2 [9]
	LGR5 [59]
	WNT2 [5]
	WNT5A [60]
	PDGFRA [61,62]

**Table 2:** Markers which expression gradients were used by [92] to isolate certain subpopulations of mesenchymal cells, and immunofluorescent staining for markers, which the authors used to identify these populations in mouse lung (presumably on E18.5).

Lung Mesenchymal Cell Types	Markers Revealed by Clustering Analysis of the Drop-Seq Data	Immunofluorescent Staining
MatrixFB-1	Tcf21, Fn1, Fgf10, and Vcam1; WNT (Wnt2, Wnt5a, and Axin2) and FGF signaling (Fgf10, Fgf7, Fgfr3, and Fgfr4); T-box TFs (Tbx2, Tbx4, and Tbx5)	Immunofluorescence staining of Fibronectin 1 (FN1), a selective marker for MatrixFB-1, was localized in peribronchiolar and perivascular fibroblasts
MatrixFB-2	Type 1 collagen (Col1a1 and Col1a2); Sfrp2, an inhibitor of WNT signaling, and a family of insulin-like growth factors and binding proteins (Igf1, Igf2, Igfbp2, and Igfbp5)	Immunofluorescence staining demonstrated a subset of MatrixFB-2 cells co-expressing SFRP2 and IGFBP5 within the mesenchymal compartment lining proximal airways
Myofibroblast-1	Expressed high levels of Pdgfra and Ednrb, but lacked mature muscle markers Actg2, Des, and Cnn1	PDGFRα-GFP^+^/αSMA^−^, absence of FN1 staining
Myofibroblast-2	Co-expressed myoFB and smooth muscle markers and may represent cells in transition from myoFBs to smooth muscle cells	PDGFRα-GFP^+^/αSMA^+^, absence of FN1 staining
Smooth muscle cells	Expressed smooth muscle markers (Actg2, Cnn1, and Des) but lacked myoFB markers Pdgfra and Ednrb	PDGFRα-GFP^−^/αSMA^+^
Pericyte-1	Pericyte selective markers, including Pdgfrb, Notch3, Mcam, Cspg4; Map3k7cl, Mustn1, and Acta2	PDGFRα-GFP^−^/αSMA^+^
Pericyte-2	Pericyte selective markers, including Pdgfrb, Notch3, Mcam, Cspg4; Agtr1a, Vsnl1, and Art3, but lacked or expressed low levels of Acta2	PDGFRβ^+^/CSPG4^+^ and PDGFRβ^+^/CSPG4^+^/ACTA2^+^

## Data Availability

Not applicable.
